# Delayed diagnosis of mild mucopolysaccharidosis type IVA

**DOI:** 10.1186/s12920-024-01910-x

**Published:** 2024-06-03

**Authors:** Mengni Yi, Pinquan Shen, Huiwen Zhang

**Affiliations:** 1 Pediatric Endocrinology and Genetics, Xinhua Hospital, Shanghai Institute for Pediatric Research, Shanghai Jiao Tong University School of Medicine, Kongjiang Road 1665 #, Shanghai, 200092 PR China; 2 Department of Pediatric Orthopaedics, Xinhua Hospital, Shanghai Jiao Tong University School of Medicine, Kongjiang Road 1665 #, Shanghai, 200092 PR China

**Keywords:** Mucopolysaccharidosis IVA, MPS IVA, GALNS, Mild type

## Abstract

**Background:**

Mucopolysaccharidosis IVA (MPS IVA) is a lysosomal storage disease caused by biallelic variants in the N-acetylgalactosamine-6-sulfatase (*GALNS*) gene and is characterized by progressive and multi-system involvements, dominantly with skeletal deformities. A mild form of MPS IVA often presents with atypical symptoms and can go unrecognized for years.

**Methods:**

The diagnosis of MPS IVA was confirmed via GALNS enzyme activity testing in leukocytes. Clinical features were collected. Molecular analysis was performed by next generation sequence and Sanger sequencing of the *GALNS* gene. The pathogenicity of the deep intron variant was verified by mRNA analyses.

**Results:**

Thirteen patients with mild MPS IVA from six families were included. All probands first visit pediatric orthopedists and it took 5.6 years to be diagnosed after the disease onset. The most common symptoms in our series were waddling gait (85%), short neck (69%) and flat feet (62%). Radiologic findings indicated skeletal abnormalities in all patients, especially modification of the vertebral bodies (100%) and acetabular and femoral head dysplasia (100%). Five novel *GALNS* variants, including c.121-2_121-1insTTTGCTGGCATATGCA, E2 deletion, c.569 A > G, c.898 + 2 T > A, and c.1139 + 2 T > C, were identified. The most common variant, a deep intron variant NM_000512.5: c.121–210 C > T (NM_001323544.2: c.129 C > T, p.G43G), was revealed to result in an 11 bp deletion (c.128_138delGCGATGCTGAG, p.Gly43Aspfs*5) on *GALNS* mRNA in the *GALNS* transcript of NM_001323544.2.

**Conclusions:**

This study provides significant insights into the clinical features and molecular characteristics that contribute to the early diagnosis of mild MPS IVA. On the basis of our cohort, orthopedists need to be able to recognize signs and symptoms of mild MPS IVA as well as the molecular and biochemical diagnosis so that an early diagnosis and treatment can be instituted.

**Supplementary Information:**

The online version contains supplementary material available at 10.1186/s12920-024-01910-x.

## Background

Mucopolysaccharidosis IVA (MPS IVA) is an autosomal recessive lysosomal storage disorder resulting from a deficiency in the activity of the enzyme N-acetylgalactosamine-6-sulfate sulfatase (GALNS) [1]. The reduced or absent GALNS enzyme activity results in the progressive accumulation of undegraded substrates, keratan sulfate (KS) and chondroitin-6-sulfate (C6S), in the lysosomes of the most tissues, which leads to multi-system manifestations, especially systemic bone dysplasia [2, 3].

MPS IVA patients exhibit a range in phenotypes, from severe, intermediate to mild [4]. Skeletal manifestations are noticed early in life in severe form of patients with MPS IVA, and severe patients often do not survive beyond the second or third decade because of pulmonary infection, cervical instability or heart valvular disease if untreated [5]. In the intermediate patients, initial symptoms may appear in early childhood. The clinical findings of these individuals progress slowly, while the disease progression will eventually develop the symptoms observed in the severe patients [4]. Among the large number of patients with MPS IVA, about 3–20% showed mild phenotype [6–8]. Symptoms may not be recognized until later in childhood in mild patients with MPS IVA, and life expectancy can be near normal [9].

*GALNS* gene (NM_000512.5) is located on chromosome 16q24.3 and has approximately 50 kb in length, organizing into 14 exons [10–12]. The *GALNS* gene is alternatively spliced, with two other known protein coding transcripts currently annotated in the Reference Sequences (RefSeq) database (NM_001323543.2 and NM_001323544.2) (https://genome.ucsc.edu/). There are 561 variants reported for the *GALNS* gene on HGMD (Human Gene Mutation Database; http://www.hgmd.org), and this high degree of genetic heterogeneity is very likely responsible for the clinical variability in patients with MPS IVA [13]. However, there were still about 6–8% mutant alleles in patients with MPS IVA that had not been identified [6, 13, 14].

MPS IVA is a progressive systemic disease that will eventually result in disability and mortality regardless of severity. Unfortunately, it may take months or even years from symptom onset to the diagnosis of MPS IVA due to the rarity of the disease, the clinical variability and the challenging differential diagnosis [2, 15]. Early diagnosis is crucial for genetic counselling and disease intervention to improve the quality of life of MPS IVA patients.

This study reported the clinical characteristics and variants of thirteen MPS IVA patients with mild type from six unrelated families, where genetic counseling was delayed as a result of the delayed diagnosis of the probands, and the family had two or more affected siblings. The knowledge of incipient clinical manifestations and novel *GALNS* variants here may assist the early diagnosis of MPS IVA disease.

## Materials and methods

### Study subjects

Patients with MPS IVA were diagnosed at the Department of Pediatric Endocrinology and Genetic Metabolism of Xinhua Hospital, affiliated to Shanghai Jiao Tong University School of Medicine, between July 2020 and August 2022. Clinical features and findings on physical examinations of patients were extracted from the electronic medical records. Standard deviation (SD) scores of weight-for-age and height-for-age for patients referred to the growth charts for Chinese children and adolescents from birth to 18 years [16]. Radiological skeletal surveys of patients were evaluated and radiologic findings were analyzed by radiologists. GALNS deficiency was demonstrated by enzyme assay [17]. Informed consent was obtained from patients or legal guardians of patients in this study. All procedures were performed under the Ethics Committee of Xinhua Hospital affiliated to Shanghai Jiao Tong University School of Medicine approval (XHEC-D-2014-006).

### Analysis of GALNS genomic DNA

Potential variants were identified by direct Sanger sequencing of 14 exons and their flanking regions of the *GALNS* gene in Family 1, 2, 5 and 6 [18]. Primers covering exon and exon–intron boundaries were assigned to produce amplicons (Table 1). Genetic analysis was performed by whole exome sequencing (WES), whole genome sequencing (WGS) and Sanger validation in Family 3. WES and Sanger sequencing of exon 2 (NM_001323544.2) of the *GALNS* gene were used to identify variants in Family 4. Genomic DNA was extracted with the DNA Blood Mini kit (Omega Bio-Tek, USA.) for cell and blood samples by a standard method following the manufacturer’s instructions. Parental DNA samples were analyzed to establish the variant origin.

**Table 1:** **Primers used for genomic PCR and amplifying cDNA**

Name	Primer sequence (5’ − 3’)	Annealing temperature (°C)	Length (bp)
NM_000512.5-E1F	CGGGGCTCCGCGGCTCCCGTGGTTG	67	171
NM_000512.5-E1R	CTGCCCCGTCCCACCGCCCGCACTC		
NM_000512.5-E2F	CCGACACGCTCTTGGCAC	61	318
NM_000512.5-E2R	AGACAAGGTTGATGCAGCCG		
NM_000512.5-E3F	TCGTCTGTCACGCGTCTGTC	61	282
NM_000512.5-E3R	CACCTGCAGCTTGCCACC		
NM_000512.5-E4F	CCTGTTAGGATGTGTGGACGC	61	334
NM_000512.5-E4R	CCAGAATCAGCTGCCGTT		
NM_000512.5-E5F	TGAAGGTGGTATCTGTTGCTGC	61	327
NM_000512.5-E5R	CATGAGTGGCGACTTGAGCC		
NM_000512.5-E6F	ATGGCTTTGCTGGTGAAATC	61	254
NM_000512.5-E6R	GGTGAGGTTGATGCATTCCT		
NM_000512.5-E7F	GACCGCACCAACCTCGCC	67	463
NM_000512.5-E7R	TGAAGGACAGAGCCAGCACC		
NM_000512.5-E8F	GGTGCTGGCTCTGTCCTTCA	61	483
NM_000512.5-E8R	TCGGTGACATCTGCTCCTCC		
NM_000512.5-E9F	GCGGGAGTGTACCTCTCTGA	64	503
NM_000512.5-E9R	GAGAGCGGTGAGGATGAGC		
NM_000512.5-E10F	TGAGGCTCCTCTGTCTCTCACA	61	437
NM_000512.5-E10R	AGCACGCCTGTGTCCAGAAC		
NM_000512.5-E11F	TGGAGGCATGAGCCACTGAT	61	315
NM_000512.5-E11R	GGAGTTCCTGCCTGTCTCACC		
NM_000512.5-E12F	CTTCAGCGTTTAGCCAGCG	58	494
NM_000512.5-E12R	ACCAAGCACGTGTGGGTATG		
NM_000512.5-E13F	GACTGCTCACTGTGGTTCTCAGC	61	366
NM_000512.5-E13R	GGCCTCACCACTGACGGAG		
NM_000512.5-E14F	AACTTGGGGAACCCTTGTCT	61	624
NM_000512.5-E14R	GTCTGCAGGTGCTGTCTGTC		
NM_001323544.2-E2F	TGGGGGACAGTTACCAGTTGA	62	353
NM_001323544.2-E2R	TGGAAGCCAGCACCACCCTGT		
NM_001323544.2-cDNA-F	CATGCACGTGTTTAGAGGC	55	614
NM_001323544.2-cDNA-R	CCAACCATCTCCCAGTCC		

### Cell culture and treatment

Epstein-Barr virus (EBV)-infected lymphoblastoid cell line from patient8 (Pt8) was established as previously reported [19, 20]. EBV-lymphoblasts were grown in RPMI 1640 medium (Gibco, USA), supplemented with 20% fetal bovine serum (FBS; Gibco, USA) and 1% penicillin/streptomycin (Invitrogen, USA). The cells were cultured at 37 °C in a humidified atmosphere with 5% CO_2_. Patient EBV-lymphoblasts were maintained in the medium with 3000 µg/ml cycloheximide (Sigma, USA) for 16 h prior to RNA extraction [21].

### RNA isolation and cDNA analysis

Total cellular RNA was isolated from EBV-lymphoblasts of Pt8 using TRIZOL (Life Technologies, USA) and then was reversely transcribed using Primer Script TM RT regent Kit (TaKaRa, Japan). Primers covering the variant NM_001323544.2: c.129 C > T and spanning at least two exons was designed (Table 1). The reverse-transcribed product was mixed with a set of primers and Go Taq Green Master mix (Promega, USA) in a final volume of 25 µL to produce a 614-bp amplicon. Then the amplicon was sequenced bidirectionally.

## Results

### Clinical findings

**Fig. 1:**
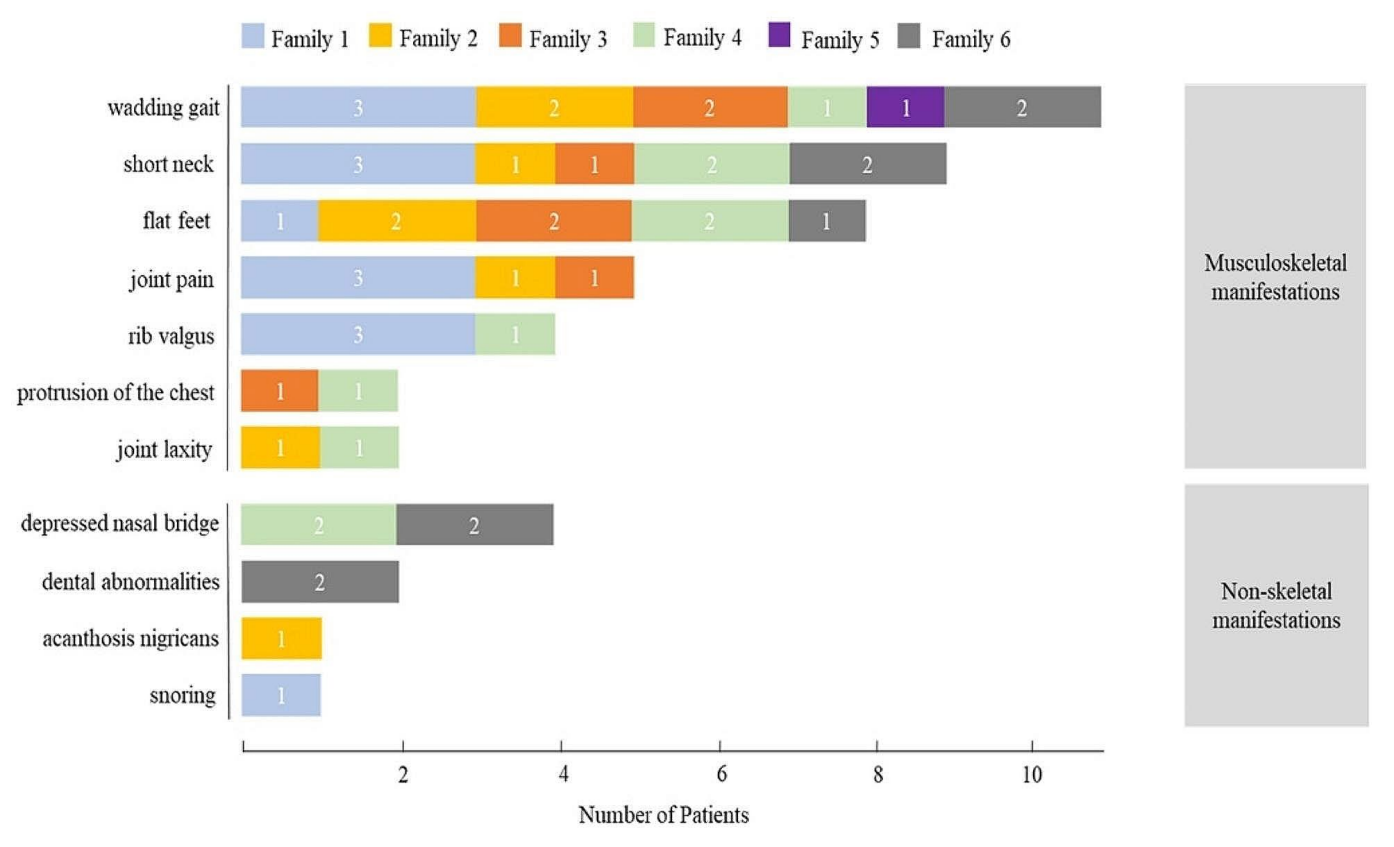
**Clinical presentation of the mild patients with MPS IVA.** The number in rectangles with different color represents the number of patients in each family

Thirteen MPS IVA patients with mild type from six unrelated families were included in this study and the male/female ratio was at 7/6. The clinical findings and biochemical characteristics of patients were summarized in Fig. 1; Table 2.

As illustrated in Fig. 1, musculoskeletal abnormalities were identified in the mild patients with MPS IVA with a decreasing order of frequency as follows: waddling gait (11/13, 85%), short neck (9/13, 69%), flat feet (8/13, 62%), joint pain (5/13, 38%), rib valgus (4/13, 31%), protrusion of the chest (2/13, 15%), and joint laxity (2/13, 15%). All probands showed waddling gait and four of them also had flat feet. A minority of mild patients presented the non-skeletal manifestations including depressed nasal bridge (4/13, 31%), dental abnormalities (2/13, 15%), acanthosis nigricans (1/13, 8%) and snoring (1/13, 8%). The median age at symptom onset for all patients was 6.3 years and at confirmed diagnosis was 10.0 years. Moreover, the median age at diagnosis (12.4 years) of probands was delayed by 5.6 years since the onset of signs and symptoms (6.8 years). The GALNS enzyme activities of these patients ranged from 1.47 to 22.40 nmol/17 h/mg protein (Table 2). All patients were normal in cognitive capability, and their parents were nonconsanguineous couples.

In this study, two patients (15%; 2/13) showed mild growth retardation at last observation, the height of these two patients corresponded to − 2.2 SD for age-matched normal controls (Table 2). No significant short stature could be observed in 85% (11/13) of patients with age ranging from 4.2 to 17 years (− 1.8 ∼ 0.7 SD). The weight of twelve patients (92%) nearly corresponded to that of the normal population (− 1.7 ∼ 0.9 SD). One patient exhibited weight lower than the age-matched weight from the growth chart of normal controls (− 2.0 SD).

**Table 2:** Clinical, and biochemical findings of mild patients with MPS IVA in this study

	Family 1			Family 2		Family 3		Family 4		Family 5		Family 6	
Patient (Pt)	1	2	3	4	5	6	7	8	9	10	11	12	13
Sex	female	male	male	male	male	female	female	male	female	male	female	female	male
**Age at onset (years)**	10.0	8.0	8.0	5.0	7.5	9.0	5.0	2.0	3.6	NA	1.0	8.0	8.0
**Age at diagnosis (years)**	13.5	9.7	9.7	10.8	8.2	17.0	9.0	12.2	3.9	7.8	6.0	13.0	9.0
**GALNS activity (nmol/17 h/mg protein)** ^**†**^	22.40	17.43	16.90	4.22	3.37	3.57	2.08	1.47	2.00	7.13	7.39	4.84	NA
**Age at last observation (years)**	14.2	10.3	10.3	12.7	10.6	17.0	9.0	13.5	4.9	9.8	6.0	14.0	10.0
**Height at last observation, cm (SD)**	151.0 (− 1.3)	141.0 (− 0.2)	147.0 (0.7)	142.0 (− 1.8))	143.0 (0.1)	150.4 (− 1.8)	132.5 (0.3)	146 (− 2.2)	109.6 (− 0.4)	133.0 (− 1.2)	106.8 (− 2.2)	150.5 (− 0.6)	129.5 (− 1.7)
**Weight at last observation, kg (SD)**	49.0 (0.1)	33.0 (− 0.5)	34.0 (− 0.3)	54.6 (0.8)	46.0 (0.9)	47.5 (− 0.7)	36.8 (0.7)	35.6 (− 2.0)	15.8 (− 1.7))	30.0 (− 0.8)	17.2 (− 1.5)	50.1 (0.2)	28.4 (− 1.2)

^†^ Normal range of GALNS activity in our laboratory is 24.44–216.69 nmol/17 h/mg protein

NA: not available

### Radiologic findings

**Fig. 2:**
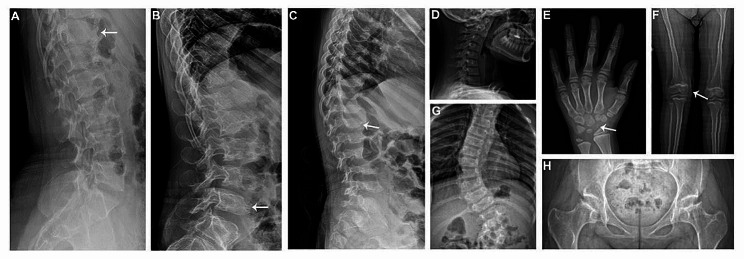
Radiographic features in mild patients with MPS IVA. Lateral lumbar spine radiographs showed irregular vertebral bodies with “tongue-shaped” deformity in Pt1 (**A**), anterior beaking of the vertebral bodies in Pt8 (**B**) and anterior sharpening of vertebral bodies in Pt9 (**C**). Radiographs revealed kyphotic cervical curve in Pt6 (**D**), dysplastic carpal bones (**E**) and right genu valgum (**F**) in Pt5, scoliosis in Pt11 (**G**) and acetabular and femoral head dysplasia in Pt12 (**H**)

Modification of the vertebral bodies, including platyspondyly, irregularity in the vertebral bodies with “tongue-shaped” deformity and anterior sharpening/beaking of the vertebral bodies, was present on skeletal radiographs of all patients (Fig. 2A-C). In the cervical spine, seven patients showed cervical kyphosis or straight cervical curve (Fig. 2D). In the hands, dysplastic carpal bones were observed in three patients and delayed bone age was noted in two patients (Fig. 2E). Genu valgum was noted in three patients (Fig. 2F). The radiographs of the spine demonstrated scoliosis in six patients (Fig. 2G). All patients showed acetabular and/or femoral head dysplasia (Fig. 2H).

### Molecular results

**Fig. 3:**
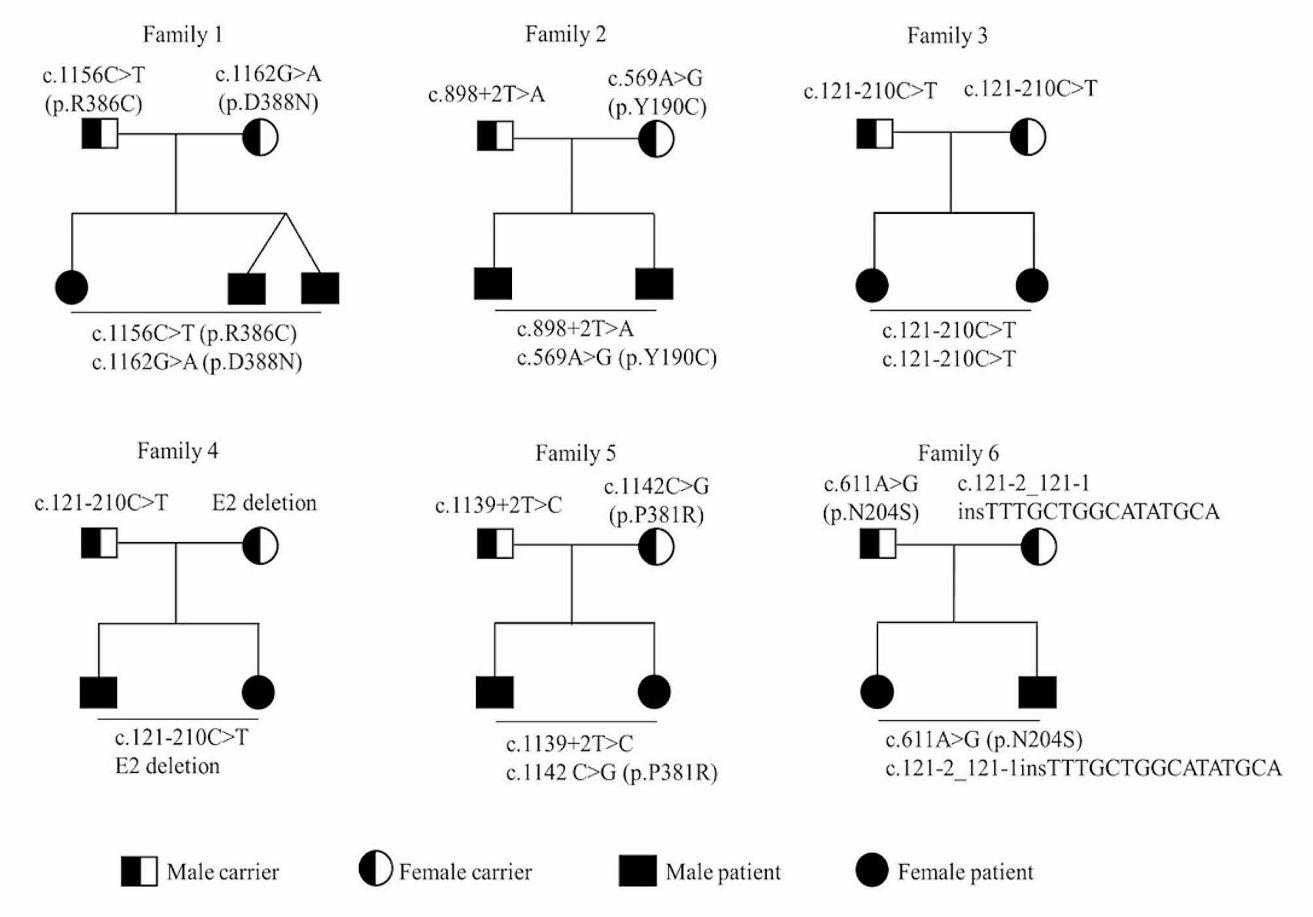
Six family trees in this study. Black-and-white rectangle represents male carrier. Black-and-white circle represents female carrier. Black rectangle represents male patient. Black circle represents female patient

Five missense variants (c.569 A > G, c.611 A > G, c.1142 C > G, c.1156 C > T, and c.1162G > A), three splice site variants (c.121–210 C > T, c.898 + 2T > A, and c.1139 + 2T > C), one small insertion (c.121-2_121-1insTTTGCTGGCATATGCA) and one large deletion (E2 deletion) were identified in thirteen individuals from six unrelated families (Additional file 1 Table S1). Five *GALNS* variants (c.121-2_121-1insTTTGCTGGCATATGCA, E2 deletion, c.569 A > G, c.898 + 2 T > A, and c.1139 + 2 T > C) were described for the first time in patients with MPS IVA. The classification of each variant was performed according to the American College of Medical Genetics and Genomics (ACMG) guidelines, and the pathogenicity of missense variants was evaluated through web-based tools (Additional file 1 Table S2 and S3). There are six genotypes, one of which was homozygous (Fig. 3). The variant NM_000512.5: c.121–210 C > T (NM_001323544.2: c.129 C > T, p.G43G) was the most prevalent, accounting for 25% of mutant alleles (3/12).

### The *GALNS* gene and mRNA molecular analyses in Family 3 and Family 4

Initial sequencing by WES and WGS in Pt6 and Pt7 from Family 3 failed to identify any disease-causing variants. However, the clinical manifestations, radiographic findings and the outcome of enzyme activity testing were strongly suggestive of MPS IVA, which led us to reanalysis of the WGS data. Finally, the deep intronic variant NM_000512.5: c.121–210 C > T, for transcript NM_001323544.2, named c.129 C > T (p.G43G), was identified in these two sisters as homozygous status by another round of bioinformatic analysis and was validated by Sanger sequencing. Their parents were carriers of this variant.

**Fig. 4:**
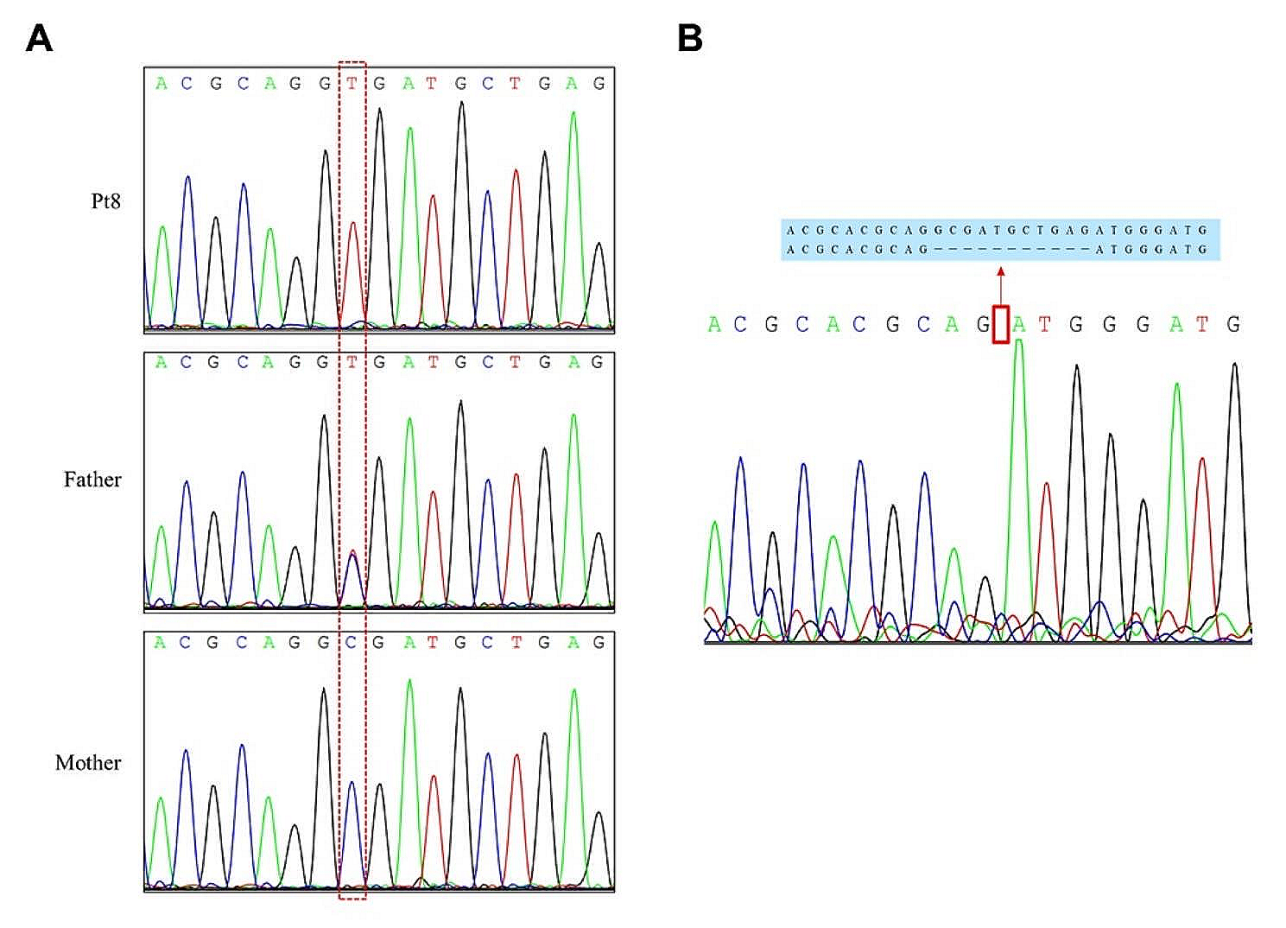
*GALNS* variants detected in the DNA and cDNA products from Pt8. The variant NM_000512.5: c.121–210 C > T (NM_001323544.2: c.129 C > T) was identified in Pt8 and his father, while the mother of Pt8 did not carry this variant (**A**). An 11 bp deletion of the *GALNS* gene was detected in cDNA from Pt8 (NM_001323544.2: c.128_138 delGCGATGCTGAG) (**B**)

For the Family 4, the results of WES and panel sequencing of 13 mucopolysaccharidosis-related genes were negative in Pt8 and Pt9. Considering that Family 3 and Family 4 were from the same province and had the same mild type of MPS IVA, we wondered whether these two families had the same *GALNS* variant. We designed the primers for exon 2 (NM_001323544.2) and performed Sanger sequencing on the amplicons. As anticipated, the variant NM_000512.5: c.121–210 C > T was identified in a homozygous status in Family 4 (Fig. 4A). However, the results of parental testing showed that only their father was carrier of the variant c.121–210 C > T and the mother did not carry it (Fig. 4A). This apparently homozygous status might result from the large deletions covering this site. Later, the second disease-causing variant, exon 2 deletion (chr16:88909113–88,909,575 deletion), was identified in two patients and mother by analysis of copy number variations (CNVs) on WES data.

The deep intron variant c.121–210 C > T in the *GALNS* transcript of NM_000512.5 is also located into the exon2 (c.129 C > T) in the *GALNS* transcript of NM_001323544.2, leading to a synonymous variant (p.G43G). To prove whether this synonymous variant causes abnormal mRNA processing, we constructed EBV-infected lymphoblastoid cell lines from Pt8 and performed cDNA analysis. The aberrant product of cDNA revealed an 11 bp deletion (c.128_138delGCGATGCTGAG) in the *GALNS* transcript of NM_001323544.2, which is predicted to result in a premature termination codon (NM_001323544.2: p.Gly43Aspfs*5) (Fig. 4B).

## Discussion

In this study, we included the clinical characteristics and *GALNS* variants in thirteen mild patients with MPS IVA from six unrelated families. Six probands mainly complained of waddling gait and visited pediatric orthopedics, which were then referred to geneticist. A definitive diagnosis of MPS IVA was demonstrated by reduced enzymatic activity of GALNS in leukocytes. Genetic analysis was performed as a confirmation of the diagnosis.

Typical MPS IVA manifests skeletal deformities, including short stature, genu valgum, pectus carinatum and spinal abnormalities [2]. The mild disease form is generally characterized by later onset of symptoms and milder symptoms, which frequently goes misdiagnosis or delayed diagnosis. In six probands here, MPS IVA diagnosis was made after 7.8 years of age (range 7.8–17 years) with the median age of 12.4 years, which was 5.6 years delayed since the disease onset. Due to delayed diagnosis genetic counseling was delayed, and the family had two or more affected siblings.

Our study showed that the pathological change was initially restricted to osteoarticular system in these mild patients with MPS IVA. Waddling gait was the most prevalent clinical feature in the mild patients with MPS IVA (11/13, 85%). Two mild patients with MPS IVA had not showed the waddling gait at last observation, which may be explained by their young age (4.2 years and 6.0 years, respectively). Other bone deformity presented in mild patients with MPS IVA mainly included short neck (9/13, 69%), flat feet (8/13, 62%) and joint pain (5/13, 38%). Flat feet were observed as a new clinical feature and had been seen in 67% of probands (4/6), indicating that the plantar performance of suspected patients should be scrutinized in the future. The evaluation of radiological findings is useful for suggesting and supporting MPS IVA. Vertebral morphologic abnormalities (13/13, 100%), acetabular and/or femoral head dysplasia (13/13, 100%), straightened cervical curvature (7/13, 54%) and scoliosis (6/13, 46%) were the most common radiological features in our series. The symptoms of non-skeletal manifestations are not obvious in these patients so far.

Short stature is a pivotal parameter in the evaluation of children with typical MPS IVA [22]. The growth of patients affected with the severe MPS IVA was reduced after two years of age and nearly stopped at approximately 7 or 8 years of age, while the patients with mild phenotype may continue to grow in their teenage years and exceed 140 cm in height [4]. In our study, the heights of eight mild patients with MPS IVA, which were above 10 years old, had been above 140 cm. Of thirteen patients with mild MPS IVA, only two patients showed mild growth retardation at last observation. These findings suggest that it cannot rule out the suspicion of MPS IVA even if there is no obvious growth retardation.

We identified ten *GALNS* variants including five missense variants, three splice-site variants, one insertion and one large deletion. Five of the variants (c.121-2_121-1insTTTGCTGGCATATGCA, E2 deletion, c.569 A > G, c.898 + 2T > A, and c.1139 + 2T > C) were described here for the first time in MPS IVA patients. According to the ACMG guidelines, four novel variants, including c.121-2_121-1insTTTGCTGGCATATGCA, E2 deletion, c.898 + 2T > A, and c.1139 + 2T > C, were categorized as ‘pathogenic’, which are supported by PVS1, PM2, PM3, PP1 and PP4. Variant c.569 A > G is categorized as ‘Likely pathogenic’, which is supported by PM2, PM3, PP1, PP4 and PP5. The other five variants (c.121–210 C > T, c.611 A > G, c.1142 C > G, c.1156 C > T and c.1162G > A) were previously reported [6, 13, 23, 24].

The variant c.121–210 C > T was classified as a variant of uncertain significance in previous study [13]. The allele frequency of this variant varies by ethnicity. In the African American population, the frequency is 0.0357 in ExAC, and 0.0297 in gnomAD, and it is 0.0333 for the African population, and 0.0043 for the Latino population in 1000 Genomes. The highest population allele frequency of variant c.121–210 C > T in other population is < 0.003 in databases including ExAC, gnomAD and 1000 Genomes, but it is zero in particular in the East Asian population. Splice Site Prediction by Neural Network (https://www.fruitfly.org/seq_tools/splice.html) predicts that c.121–210 C > T may cause a splice donor site. Variants located within deep intronic regions appear capable of promoting the use of alternative splicing sites. Therefore, the variant NM_000512.5: c.121–210 C > T (NM_001323544.2: c.129 C > T; p.G43G) contributes to the clinical phenotype in these individuals by causing abnormal mRNA processing. To prove this prediction, RNA was isolated from Family 4 and cDNA analysis was performed. Considering that the variant NM_001323544.2: c.129 C > T (p.G43G) may result in a premature termination codon, and initiate degradation of mutant transcripts through the nonsense-mediated messenger RNA (mRNA) decay (NMD) pathway, we used cycloheximide as a NMD inhibitor in cultured patient EBV-lymphoblasts to stabilize the unstable transcripts [21, 25]. Finally, an 11 bp deletion (NM_001323544.2: c.128_138del) was identified in cDNA of Pt8, which introduced a premature termination codon at 5 amino acids later after the first amino acid change (p.Gly43Aspfs*5). This abnormal splicing event strongly supported the clinical phenotype observed in the patient with variant c.121–210 C > T.

As shown above, mild phenotype of MPS IVA should be considered in the presence of waddling gait, short neck, flat feet and joint pain involvement, and the absence of typical features does not rule out MPS IVA. Radiographic findings such as vertebral morphologic abnormalities, scoliosis and acetabular and/or femoral head dysplasia may indicate MPS IVA. For orthopedists who may not be familiar with MPS IVA, it is necessary to collaborate with a physician familiar with the diagnosis of inherited metabolic disorders such as a clinical geneticist or a metabolic specialist. Moreover, molecular diagnosis route here suggests that deep intronic variants may be responsible for occasional unidentified *GALNS* disease alleles and parental genotype should be studied especially for homozygous variants along the MPS IVA diagnostic process.

## Conclusions

On the basis of our cohort, increased awareness of mild MPS IVA among orthopedists is needed. If findings indicating MPS IVA are present, evaluation of enzyme activity will be the best option, along with genetic testing. Furthermore, deep intronic variants should be taken seriously in the genetic analysis process, especially in whom the disease-causing *GALNS* variant was not initially identifiable by next generation sequence.

### Electronic supplementary material

Below is the link to the electronic supplementary material.

Supplementary Material 1

## Data Availability

The datasets generated and/or analysed during the current study are available in the clinVAR repository, [accession numbers SCV004697574-SCV004697582 and SCV004697851].
