# Supplementary material for: Delayed diagnosis of mild mucopolysaccharidosis type IVA
Source: BMC Med Genomics. 2024 Jun 3;17:151. doi: 10.1186/s12920-024-01910-x (PMC11145893; doi:10.1186/s12920-024-01910-x)
Supplement: Supplementary file 1 — Supplementary Material 1 [file 12920_2024_1910_MOESM1_ESM.docx]

**Table S1. Variants in the *GALNS* gene causing MPS IVA in this study**

| **Exon** | **Nucleotide change** | **Predicted effect on protein** | **Alleles reported** | **Ethnicity** | **reference** |
| --- | --- | --- | --- | --- | --- |
| intron 1 | c.121-210C>T | c.121-210C>T | 3 | unknown | [1] |
| intron 1 | c.121-2_121-1 insTTTGCTGGCATATGCA | c.121-2_121-1 insTTTGCTGGCATATGCA | 1 | Chinese | this study |
| E2 | E2 deletion | E2 deletion | 1 | Chinese | this study |
| E6 | c.569A>G | p.Y190C | 1 | Chinese | this study |
| E6 | c.611A>G | p.N204S | 1 | Chinese | [2] |
| intron 8 | c.898+2T>A | c.898+2T>A | 1 | Chinese | this study |
| intron 10 | c.1139+2T>C | c.1139+2T>C | 1 | Chinese | this study |
| E11 | c.1142C>G | p.P381R | 1 | Chinese | [3] |
| E11 | c.1156C>T | p.R386C | 1 | American Caucasian, American Caucasian: Hispanic/Greek, American Caucasian: Italian, Argentine, Brazilian, British, Canadian Caucasian, Chilean, Chinese, Colombian, French, German, Italian, Japanese, Korean, Mexican, Polish, Portuguese, Spanish, Turkish, | [3-16] |
| E11 | c.1162G>A | p.D388N | 1 | Italian, unknown | [3, 15, 16] |

**Table S2. ACMG rating of novel variants in this study.**

| **nucleotide change** | **Predicted effect on protein** | **Position (hg19)** | **ACMG rating^†^** | **PVS1** | **PS1** | **PS2** | **PS3** | **PS4** | **PM1** | **PM2** | **PM3** | **PM4** | **PM5** | **PM6** | **PP1** | **PP2** | **PP3** | **PP4** | **PP5** | **BA1** | **BS1** | **BS2** | **BS3** | **BS4** | **BP1** | **BP2** | **BP3** | **BP4** | **BP5** | **BP6** | **BP7** |
| --- | --- | --- | --- | --- | --- | --- | --- | --- | --- | --- | --- | --- | --- | --- | --- | --- | --- | --- | --- | --- | --- | --- | --- | --- | --- | --- | --- | --- | --- | --- | --- |
| c.121-2_121-1 insTTTGCTGGCATATGCA | c.121-2_121-1 insTTTGCTGGCATATGCA | chr16:88909238 | P | 1 | 0 | 0 | 0 | 0 | 0 | 1 | 1 | 0 | 0 | 0 | 1 | 0 | 0 | 1 | 0 | 0 | 0 | 0 | 0 | 0 | 0 | 0 | 0 | 0 | 0 | 0 | 0 |
| E2 deletion | E2 deletion | chr16:88909113-  88909575 | P | 1 | 0 | 0 | 0 | 0 | 0 | 1 | 1 | 0 | 0 | 0 | 1 | 0 | 0 | 1 | 0 | 0 | 0 | 0 | 0 | 0 | 0 | 0 | 0 | 0 | 0 | 0 | 0 |
| c.569A>G | p.Y190C | chr16:88902673 | LP | 0 | 0 | 0 | 0 | 0 | 0 | 1 | 1 | 0 | 0 | 0 | 1 | 0 | 1 | 1 | 0 | 0 | 0 | 0 | 0 | 0 | 0 | 0 | 0 | 0 | 0 | 0 | 0 |
| c.898+2T>A | c.898+2 T>A | chr16:88901619 | P | 1 | 0 | 0 | 0 | 0 | 0 | 1 | 1 | 0 | 0 | 0 | 1 | 0 | 0 | 1 | 0 | 0 | 0 | 0 | 0 | 0 | 0 | 0 | 0 | 0 | 0 | 0 | 0 |
| c.1139+2T>C | c.1139+2 T>C | chr16:88893108 | P | 1 | 0 | 0 | 0 | 0 | 0 | 1 | 1 | 0 | 0 | 0 | 1 | 0 | 0 | 1 | 0 | 0 | 0 | 0 | 0 | 0 | 0 | 0 | 0 | 0 | 0 | 0 | 0 |

^†^P: Pathogenic; LP: Likely pathogenic.

**Table S3. Potential pathogenicity analyses of novel missense variant using web-based tools.** Predictions for the pathogenicity of missense variants were obtained based on results of SIFT, Mutation Taster, PolyPhen-2, MutationTaster, Mendelian Clinically Approved Pathogenicity (M-CAP), Genomic Evolutionary Rate Profiling (GERP++), as well as population databases, such as the genome Aggregation Database (gnomAD), Exome Aggregation Consortium (ExAC), and 1000Genomes.

| nucleotide change | protein alteration | SIFT_pred/score | Polyphen2_HDIV_pred/score | Polyphen2_HVAR_pred/score | MutationTaster_pred/score | M-CAP_pred/score | GERP++_pred/score | gnomAD | ExAC | 1000Genomes |
| --- | --- | --- | --- | --- | --- | --- | --- | --- | --- | --- |
| c.569A>G | p.Y190C | Damaging/0.042 | Probably_damaging/0.998 | Probably_damaging/0.926 | Disease_causing/1 | Damaging/0.377 | Nonconserved/-0.067 | - | - | - |

**REFERENCES**

1. Zanetti A, D'Avanzo F, AlSayed M, Brusius-Facchin AC, Chien YH, Giugliani R, et al. Molecular basis of mucopolysaccharidosis IVA (Morquio A syndrome): A review and classification of GALNS gene variants and reporting of 68 novel variants. Hum Mutat. 2021;42:1384-1398.

2. Ficicioglu C, Matalon DR, Luongo N, Menello C, Kornafel T, and Degnan AJ. Diagnostic journey and impact of enzyme replacement therapy for mucopolysaccharidosis IVA: a sibling control study. Orphanet J Rare Dis. 2020;15:336.

3. Yi M, Wang Y, Gao X, Han L, Qiu W, Gu X, et al. Investigation of GALNS variants and genotype-phenotype correlations in a large cohort of patients with mucopolysaccharidosis type IVA. J Inherit Metab Dis. 2022;45:593-604.

4. Bunge S, Kleijer WJ, Tylki-Szymanska A, Steglich C, Beck M, Tomatsu S, et al. Identification of 31 novel mutations in the N-acetylgalactosamine-6-sulfatase gene reveals excessive allelic heterogeneity among patients with Morquio A syndrome. Hum Mutat. 1997;10:223-32.

5. Dũng VC, Tomatsu S, Montaño AM, Gottesman G, Bober MB, Mackenzie W, et al. Mucopolysaccharidosis IVA: correlation between genotype, phenotype and keratan sulfate levels. Mol Genet Metab. 2013;110:129-38.

6. Fukuda S, Tomatsu S, Cooper A, Wraith JE, Kato Z, Yamada N, et al. Mucopolysaccharidosis IVA (Morquio A): three novel small deletions in the N-acetylgalactosamine-6-sulfate sulfatase gene. Hum Mutat. 1996;8:187-90.

7. He D, Huang Y, Ou Z, Sheng H, Li S, Zhao X, et al. Molecular genetic assay of mucopolysaccharidosis IVA in South China. Gene. 2013;532:46-52.

8. Lee NH, Cho SY, Maeng SH, Jeon TY, Sohn YB, Kim SJ, et al. Clinical, radiologic, and genetic features of Korean patients with Mucopolysaccharidosis IVA. Korean J Pediatr. 2012;55:430-7.

9. Morrone A, Tylee KL, Al-Sayed M, Brusius-Facchin AC, Caciotti A, Church HJ, et al. Molecular testing of 163 patients with Morquio A (Mucopolysaccharidosis IVA) identifies 39 novel GALNS mutations. Mol Genet Metab. 2014;112:160-70.

10. Ogawa T, Tomatsu S, Fukuda S, Yamagishi A, Rezvi GM, Sukegawa K, et al. Mucopolysaccharidosis IVA: screening and identification of mutations of the N-acetylgalactosamine-6-sulfate sulfatase gene. Hum Mol Genet. 1995;4:341-9.

11. Pajares S, Alcalde C, Couce ML, Del Toro M, Gonzalez-Meneses A, Guillen E, et al. Molecular analysis of mucopolysaccharidosis IVA (Morquio A) in Spain. Mol Genet Metab. 2012;106:196-201.

12. Park HD, Ko AR, Ki CS, Lee SY, Kim JW, Cho SY, et al. Five novel mutations of GALNS in Korean patients with mucopolysaccharidosis IVA. Am J Med Genet A. 2013;161A:509-17.

13. Tomatsu S, Fukuda S, Cooper A, Wraith JE, Ferreira P, Di Natale P, et al. Fourteen novel mucopolysaccharidosis IVA producing mutations in GALNS gene. Hum Mutat. 1997;10:368-75.

14. Tomatsu S, Nishioka T, Montaño AM, Gutierrez MA, Pena OS, Orii KO, et al. Mucopolysaccharidosis IVA: identification of mutations and methylation study in GALNS gene. J Med Genet. 2004;41:e98.

15. Tomatsu S, Montaño AM, Nishioka T, Gutierrez MA, Pena OM, Tranda Firescu GG, et al. Mutation and polymorphism spectrum of the GALNS gene in mucopolysaccharidosis IVA (Morquio A). Hum Mutat. 2005;26:500-12.

16. Wang Z, Zhang W, Wang Y, Meng Y, Su L, Shi H, et al. Mucopolysaccharidosis IVA mutations in Chinese patients: 16 novel mutations. J Hum Genet. 2010;55:534-40.
